# Efficacy of supramalleolar osteotomy in the treatment of traumatic ankle joint varus deformity in adolescents

**DOI:** 10.1186/s13018-023-04239-w

**Published:** 2023-10-04

**Authors:** Bo Zhao, Wei Liu, Yaqiong Zhao, Paerhati Wahafu, Xue Wang, Ling Qi, Chengwei Wang

**Affiliations:** 1 The Sixth Affiliated Hospital of Xinjiang Medical University, Urumqi, Xinjiang 830000 People’s Republic of China; 2 The Affiliated Tumor Hospital of Xinjiang Medical University, Urumqi, Xinjiang 830000 People’s Republic of China

**Keywords:** Adolescent, Supramalleolar osteotomy, Traumatic ankle varus, Realignment surgery

## Abstract

**Background:**

Supramalleolar osteotomy (SMOT) has emerged as a valuable treatment for ankle varus deformity; however, there are fewer reports of treatment outcomes in adolescents. The purpose of this study was to investigate the radiologic and clinical outcomes of SMOT for the treatment of traumatic ankle joint varus deformity (TAVD) in adolescents.

**Methods:**

We reviewed 32 adolescent cases who underwent SMOT between February 2017 and February 2022 for TAVD. Radiologic assessment included tibial anterior surface angle (TAS), talar tilt angle (TT), and tibial lateral surface angle (TLS) preoperatively and at 3 months and 12 months postoperatively, and clinical assessment was performed using American Orthopaedic Foot and Ankle Society (AOFAS) scores, Visual Analogue Scale (VAS) scores, and ankle dorsiflexion–plantarflexion ROM including preoperative and 6 months postoperative and 12 months postoperative.

**Results:**

All 32 patients were followed up completely with a mean follow-up of (20.3 ± 3.2) months. From the radiologic outcomes, the mean preoperative TAS improved from 61.53 ± 3.74 to 88 ± 1.72 at 12 months postoperatively, the mean preoperative TT decreased from 2.25 ± 1.32 to 0.5 ± 0.57 at 12 months postoperatively, the mean preoperative TLS improved from 76.72 ± 0.21 to 79.34 ± 1.52 at 12 months postoperatively, the differences between the above preoperative and 12 months postoperative radiologic outcomes were statistically significant (*p* < 0.05), the mean preoperative AOFAS score improved from 65.5 ± 9.40 to 92.34 ± 4.00 at 12 months postoperatively, the mean preoperative VAS score decreased from 2.44 ± 1.24 to 0.78 ± 0.75 at 12 months postoperatively, and the mean preoperative range of motion (ROM) of ankle improved from 50.16 ± 7.46 to 55.78 ± 4.77 at 12 months postoperatively. The differences between the above preoperative and 12 months postoperative clinical results were statistically significant (*p* < 0.05).

**Conclusion:**

Our study demonstrated that SMOT was effective in correcting TAVD and significantly improving ankle function in adolescents, and that it is an efficient and successful method for restoring ankle joint congruence and normal hindfoot alignment.

## Introduction

Adolescent ankle fractures are relatively common in clinical practice, and distal tibial metaphyseal fractures are a relatively common metaphyseal injury, the second most common injury after distal radial metaphyseal, accounting for approximately 11–20% of all metaphyseal fractures [1]. Adolescents are still in the developmental stage, and damage to the distal tibial epiphysis may result in developmental deformities leading to ankle varus and valgus deformities, followed by ankle instability, cartilage destruction, ankle arthritis, and other injuries, which gradually aggravate the deformities and form a vicious circle, leading to progressive aggravation of ankle dysfunction and pain, and affecting the adolescents' development and sports; compared to other lower extremity arthritis, ankle arthritis develops at a younger age and progresses more rapidly, progressing to end stage within 10–20 years of injury [2]. Therefore, the treatment of traumatic ankle joint varus deformity (TAVD) in adolescents is an urgent issue in clinical work.

Currently, the mainstay of treatment for ankle inversion deformity is joint-preserving surgery, and for patients with severe end-stage ankle arthritis, ankle arthrodesis and ankle arthroplasty are the main surgical modalities, while the more common surgical option for joint-preserving surgery is supramalleolar osteotomy (SMOT) [3]. SMOT improves the biomechanics of the ankle joint primarily by correcting the abnormal angulation of the distal tibia and adjusting the lower extremity joints [4], thereby slowing the progression and development of ankle arthritis and even reversing the radiographic staging of ankle arthritis [5]. Due to the fact that SMOT can correct ankle inversion deformities without sacrificing the ankle joint, they are significant in the treatment of TAVD in more youthful patients [6].

To the best of our knowledge, SMOT has been shown to be effective in the correction of varus and valgus deformities of the ankle in adults and in the treatment of ankle arthritis [7, 8], and it helps improve patients' quality of life [9]; nevertheless, there are not many reports on the efficacy of SMOT for the treatment of TAVD in adolescents at the developmental stage; therefore, the aim of this study was to retrospectively analyze the clinical data on the application of SMOT for the treatment of TAVD in adolescents at our institution and to evaluate the radiological and clinical outcomes of SMOT for the treatment of TAVD in adolescents.

## Patients and methods

### Patient characteristics

This was a retrospective study approved by the Ethics Committee of the Sixth Affiliated Hospital of Xinjiang Medical University, and the study protocol was conducted in accordance with the Declaration of Helsinki, with all patients providing written informed consent. In this study, we reviewed 32 cases of adolescents, 17 males and 15 females, who underwent SMOT for TAVD between February 2016 and February 2022, and the mean age of the patients at the time of surgery was 14.9 ± 1.5 years (range: 12–17 years). The inclusion exclusion criteria are shown in Table 1. Routine bipedal radiographs were taken before and after surgery, and the imaging and clinical data of all patients were examined by two senior orthopedic surgeons who were not involved in the surgery, and all patients were followed up completely.

**Table 1:** The inclusion and exclusion criteria

The inclusion criteria	The exclusion criteria
Age range 12–18 years	Congenital deformities, rheumatoid arthritis, Charcot type joints, tumors and bone metastases, bone infections
Had a distal tibiofibular fracture resulting in ankle inversion deformity ≥ 15°, disease duration > 6 months, joint deformity, dysfunction	Severe congenital underlying diseases
TAS < 75°, TT < 3°	Takakura Stage IV patients
Patient's willingness to undergo surgical treatment and follow-up of joint function	Presence of severe muscular, vascular, or neurological disease in the affected limb

TAS tibial anterior surface angle, TT talar tilt angle

## Operative technique

All of the procedures were performed in the supine position by the same surgeons under spinal or general anesthesia; a tourniquet is applied to the thigh and is used for this procedure. A 6–8 cm medial ankle incision was made while ensuring the protection of the saphenous vein and saphenous nerve. The medial tibia was then detached, and the precise position of the oblique osteotomy was determined using two parallel Kirschner wires under C-arm fluoroscopy. The plane and tilt angle of the osteotomy were calculated based on preoperative radiographs, specifically by measuring the center of rotation of angulation (CORA) of the tibia. Minimally invasive osteotome drilling was performed by creating rows of holes using a bone knife, ensuring that the osteotomy did not involve the articular surface. The tibia was gradually elevated using the stacked knife method while monitoring the tightness of the ankle joint tube. Intraoperative fluoroscopy indicated tibial anterior surface angle (TAS) to be approximately 85°-95° and tibial lateral surface angle (TLS) about 78°-83°, which helped determine the line of force in the ankle joint. Any bony cribriforms hindering the placement of the plate and impeding movement were removed. Autologous iliac bone was utilized for implantation at the osteotomy site. A distal tibia medial anatomical locking plate was then positioned for fixation, followed by suturing of the incision, placement of a drainage tube, and bandaging for stabilization. During the surgery, if the ankle canal is identified to be tight, prelaxation is performed as a preventive measure against ankle canal syndrome. In cases of intraoperative ankle displacement, lateral ankle mismatch, or impingement, fibula amputation is conducted to ensure the integrity of the coin sign, followed by immobilization using an anatomical plate. The need for adjustments to the medial and lateral ligaments is determined through stability testing of the ankle after immobilization [10].

## Postoperative treatment

The drain was removed within 48 h after surgery, and the stitches were removed at 14 days; 2–3 weeks of immobilization and braking in a cast or ankle mobility brace, 3–4 weeks of passive weightless plantarflexion and dorsiflexion, 5–7 weeks of active and passive functional exercises in all directions of the unweighted joint with the removal of the brace, and 8–12 weeks of partially weight-bearing activities under the protection of an inflatable brace. After bone healing at the osteotomy site was confirmed by clinical and imaging data, 5–10 kg per week was increased to full weight-bearing starting at 15 kg and a rehabilitation program was initiated, including a gradual return to full activity. Internal fixation plates were removed 1 to 2 years after surgery, depending on the healing of the osteotomy site, at a mean of (12.2 ± 2.1) months.

## Radiographic evaluation

Radiographic and clinical evaluations preoperatively, in the immediate postoperative period, at 1, 2, 3, 6, 12, and 18 months, and annually thereafter, at each follow-up visit, anterior posterior and lateral radiographs of the foot and ankle were performed, and TAS—the angle between the tibial axis and the articular surface of the distal tibia, and talar tilt angle (TT)—the angle between the articular surface of the distal tibia and the tangent line to the upper surface of the talus, were assessed on anterior posterior radiographs of the foot, and TLS—the angle between the tibial axis and the line joining the anterior and posterior margins of the distal tibial joint, was assessed on lateral foot radiographs, osteotomy fusion was defined as satisfying clinical criteria (no pain, no warmth, improvement in swelling, and stability to stress) and radiographic criteria (visible trabecular bridging across the osteotomy site and no lucency around the hardware) [11]. Radiographic and clinical data of all patients were randomized by two senior orthopedic surgeons who were not involved in the surgery; each surgeon was unaware of any other data on all patients. Previous studies have shown that the angular measurements used in this study have good reliability [12–14].

## Clinical evaluation

Clinical evaluation was performed using the Visual Analogue Scale (VAS) and the American Orthopaedic Foot and Ankle Society (AOFAS) scores to evaluate the functional recovery of the patients. The AOFAS scoring system consists of nine areas: pain, maximum walking distance, function, walking surface, sagittal plane motion, gait abnormalities, ankle hindfoot stability, hindfoot motion, and alignment. The VAS is performed by means of a 10-cm line, with 0 on the left indicating "no pain" and 10 on the right indicating "most pain," and manual measurement of ankle dorsiflexion–plantarflexion range of motion (ROM) using a standard protractor in a standardized sitting position [15].

## Statistical analysis

Statistical analyses were performed by SPSS version 27.0 software (SPSS Inc., Chicago, IL, USA), and all data were expressed as mean ± standard deviation, and normal and non-normal data were compared using paired one-way ANOVA and Wilcoxon signed-rank test, respectively. TAS, TT, and TLS at preoperative, 3-month postoperative, and 12-month postoperative follow-ups, and AOFAS scores, VAS scores, and ROM of ankle at preoperative, 6-month postoperative, and 12-month postoperative follow-ups were compared, and the difference was considered to be statistically significant at a *p*-value < 0.05.

## Results

### Radiologic Outcomes

**Fig. 1:**
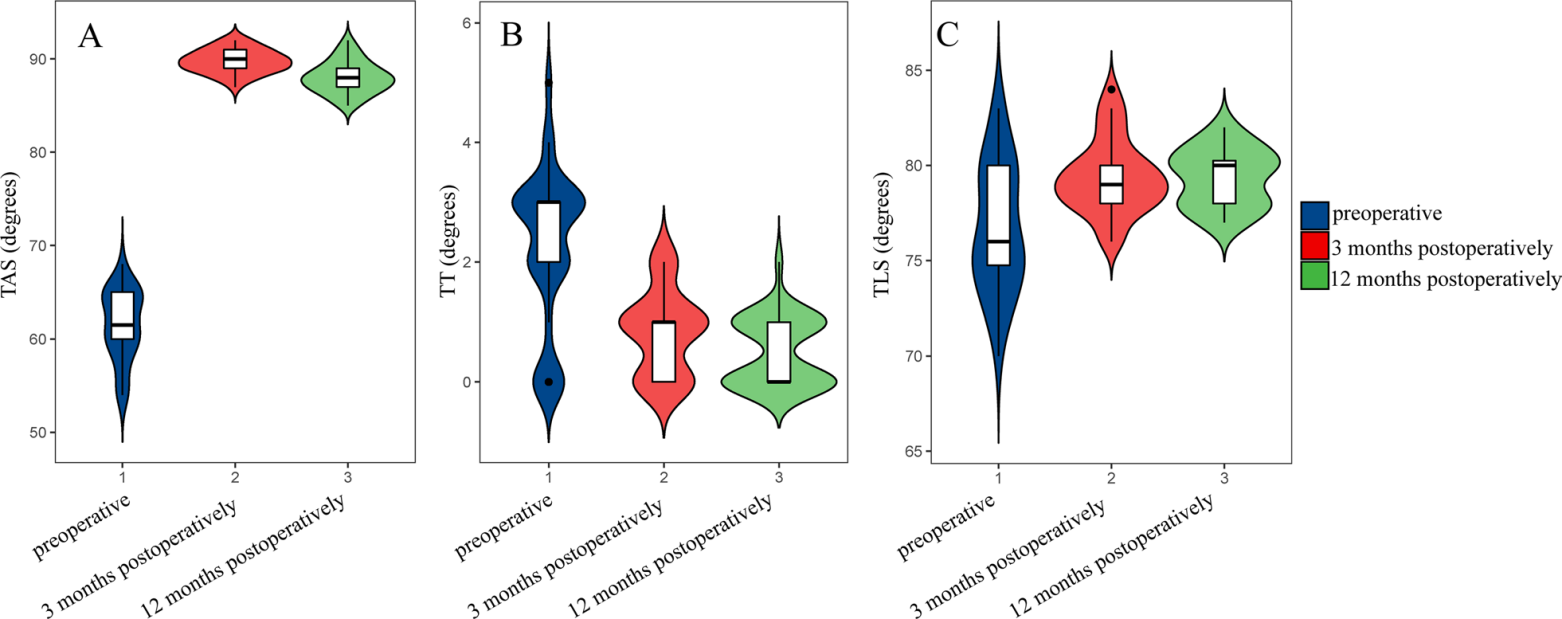
Distribution of various radiologic outcomes at different follow-up times. **A** is TAS at different follow-up times, **B** is TT at different follow-up times, and **C** is TLS at different follow-up times

The mean duration of surgery was 90 min (range: 75–120 min). All cases were followed up completely and the osteotomy sites were fused within the follow-up time (20.3 ± 3.2 months), by observing a positive correlation between healing and bracing angle, good stable ankles were obtained in all cases and remained stable at final follow-up, and the assessment showed improvement in all cases. From the radiographic outcomes, the mean preoperative TAS improved from 61.53 ± 3.74° to 88 ± 1.72° at 12 months postoperatively, which was a significant improvement, with a statistically significant difference (*p* < 0.05), the mean preoperative TT decreased from 2.25 ± 1.32° to 0.5 ± 0.57° at 12 months postoperatively, which was a significant decrease, with a statistically significant difference (*p* < 0.05), and the mean preoperative TLS improved from 76.72 ± 0.21° to 79.34 ± 1.52° at 12 months postoperatively, a statistically significant difference (*p* < 0.05). We also counted the radiographic outcomes at 3 months postoperatively, as shown in Table 2 and Fig. 1.

**Table 2:** Statistical results of two-by-two comparisons of radiologic outcomes under different follow-up time conditions

Radiologic outcome	Evaluation time	Average ± SD	Bonferroni method to compare *p* values		
			Preoperative	3 months postoperatively	12 months postoperatively
TAS (degrees)	Preoperative	61.53 ± 3.74	–	< 0.05	< 0.05
	3 months postoperatively	89.75 ± 1.27		–	< 0.05
	12 months postoperatively	88 ± 1.72			–
TT (degrees)	Preoperative	2.25 ± 1.32	–	< 0.05	< 0.05
	3 months postoperatively	0.81 ± 0.69		–	0.368
	12 months postoperatively	0.5 ± 0.57			–
TLS (degrees)	Preoperative	76.72 ± 0.21	–	< 0.05	< 0.05
	3 months postoperatively	79.38 ± 1.95		–	0.999
	12 months postoperatively	79.34 ± 1.52			–

TAS tibial anterior surface angle, TT talar tilt angle, TLS tibial lateral surface angle, SD Standard Deviation

## Clinical results

**Fig. 2:**
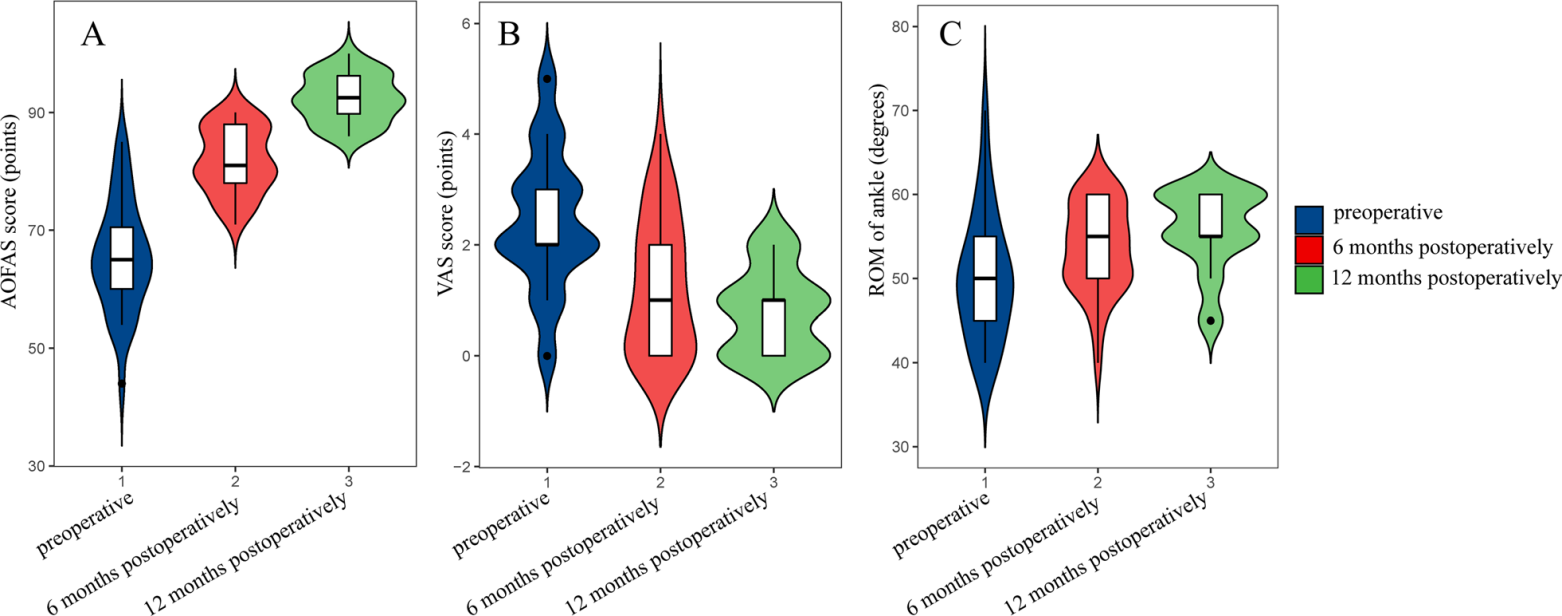
Distribution of various clinical results at different follow-up times. **A** is the AOFAS score at different follow-up times, **B** is the VAS score at different follow-up times, and **C** is the ROM of ankle at different follow-up times

**Fig. 3:**
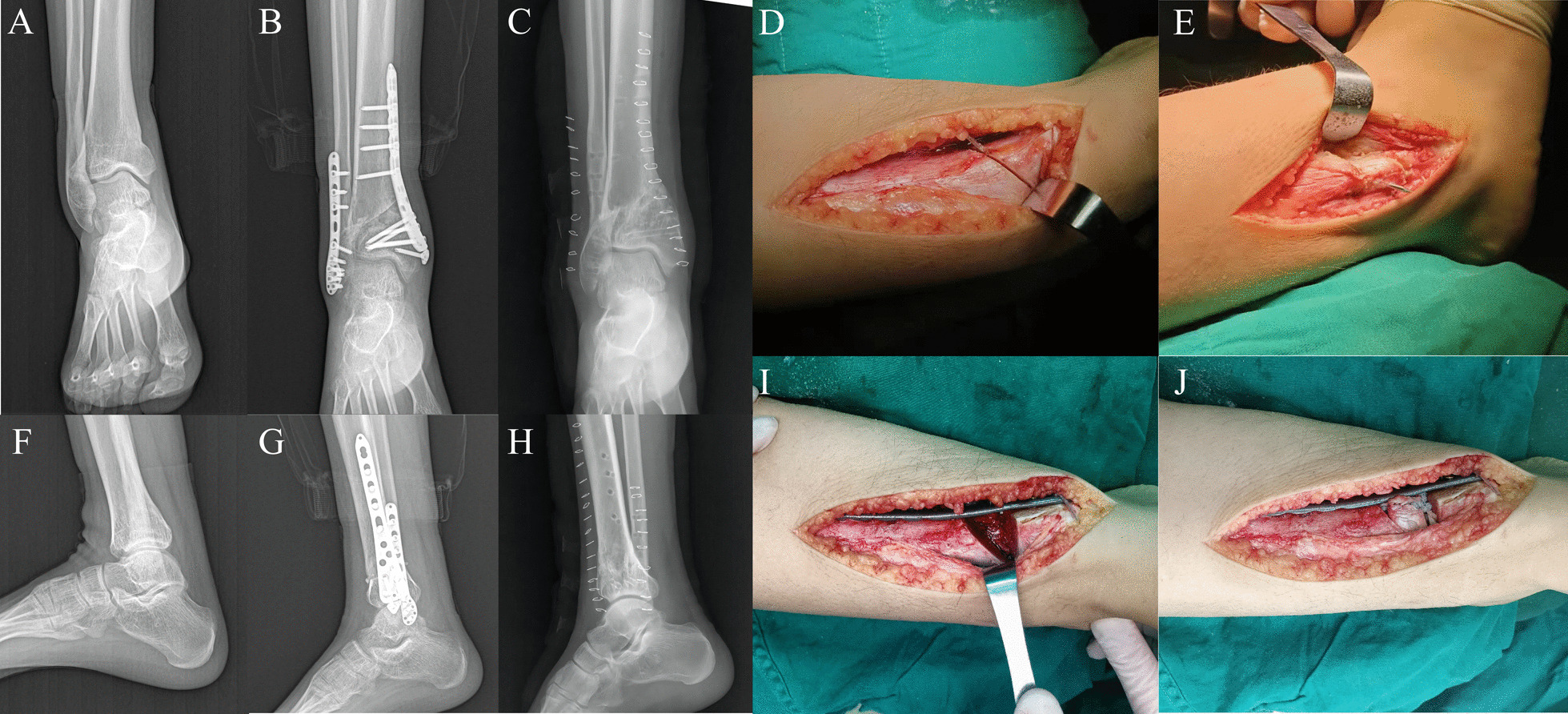
Radiographic follow-up and intraoperative photographs of a 16-year-old patient who underwent Supramalleolar osteotomy for treatment of traumatic ankle joint varus deformity. **A** is a preoperative anterior posterior radiograph, **B** is a 3-month postoperative anterior posterior radiograph, **C** is a 12-month postoperative anterior posterior radiograph, **D** is a medial ankle incision and osteotomy based on preoperative planning, **E** is a fibula osteotomy, **F** is a preoperative lateral radiograph, **G** is a 3-month postoperative lateral radiograph, **H** is a 12-month postoperative lateral radiograph, **I** is to spread the osteotomy surface and check that the distal tibia medial anatomic locking plate can be placed successfully, and **J** is the use of autogenous bone implantation at the osteotomy site followed by placement of a distal tibia medial anatomical locking plate for fixation

In terms of clinical results, the mean preoperative AOFAS score improved from 65.5 ± 9.40 to 92.34 ± 4.00 at 12 months postoperatively, getting a significant improvement, with a statistically significant difference (*p *< 0.05), and the mean preoperative VAS score decreased from 2.44 ± 1.24 to 0.78 ± 0.75 at 12 months postoperatively, getting a significant decrease, with a The difference was statistically significant (*p* < 0.05), and the mean preoperative ROM of ankle improved from 50.16 ± 7.46 to 55.78 ± 4.77 at 12 months postoperatively, which got a significant improvement, and the difference was statistically significant (*p* < 0.05). We also statistically analyzed the clinical results at 6 months postoperatively, which are shown in Table 3 and Fig. 2, and the ankle joints of all the cases were painful and functionally improved after the operation, relieved and function improved, as shown in Fig. 3.

**Table 3:** Statistical results of two-by-two comparisons of clinical results under different follow-up time conditions

Clinical results	Evaluation time	Average ± SD	Bonferroni method to compare *p* values		
			Preoperative	6 months postoperatively	12 months postoperatively
AOFAS score (points)	Preoperative	65.5 ± 9.40	–	< 0.05	< 0.05
	6 months postoperatively	81.75 ± 5.49		–	< 0.05
	12 months postoperatively	92.34 ± 4.00			–
VAS score (points)	Preoperative	2.44 ± 1.24	–	< 0.05	< 0.05
	6 months postoperatively	1.25 ± 1.22		–	0.206
	12 months postoperatively	0.78 ± 0.75			–
ROM of ankle (degrees)	Preoperative	50.16 ± 7.46	–	0.059	< 0.05
	6 months postoperatively	53.59 ± 5.27		–	0.310
	12 months postoperatively	55.78 ± 4.77			–

VAS Visual Analogue Scale, AOFAS American Orthopaedic Foot and Ankle Society, ROM range of motion, SD standard deviation

## Complications

During the observation period, no persistent bleeding occurred at the incision in all patients, one (3%) patient had incision infection, two (6%) patients had delayed healing of the incision, which was resolved by local wound care and antibiotic treatment, and one (3%) patient had postoperative popliteal vein thrombosis, which was stabilized by application of anticoagulation therapy. All patients did not require secondary surgery.

## Discussion

This study discusses the radiologic and clinical outcomes of SMOT for the treatment of TAVD in adolescents. Traumatic factors are responsible for up to 80% of ankle arthritis [16], and the prevalence of ankle osteoarthritis is higher in young people compared to patients with hip and knee arthritis. If TAVD in adolescents is not treated as early as possible, it will lead to long-term uneven loading of the talus surface, resulting in damage to the articular cartilage and ultimately osteoarthritis of the ankle joint, which is more likely to occur due to the smaller weight-bearing area of the ankle joint and the greater loading per unit area [17]; end-stage ankle arthritis can only be treated by ankle arthrodesis and ankle arthroplasty [18, 19]; therefore, TAVD in adolescents should be corrected as soon as possible by surgical treatment. SMOT may balance internal ankle stresses by shifting weight-bearing concentrated medially to the lateral side [20], thereby improving ankle biomechanics, preventing the development of ankle osteoarthritis or alleviating the pain or effects of inversion-type ankle osteoarthritis and slowing the progression of ankle osteoarthritis.

SMOT is commonly used for ankle-preserving treatment of early to mid-stage ankle osteoarthritis, and most clinical studies of supra-ankle osteotomies have focused on adult patients with ankle osteoarthritis with little attention paid to adolescents; however, there is a close relationship between ankle deformity and the development of ankle osteoarthritis [21]. Kim et al. [22] treated 13 patients with valgus ankle osteoarthritis with a mean age of 51.2 years (29 ~ 69 years) with SMOT and followed up for up to 2 years, which showed improvement in arthritis grading in all but one patient, all of whom did not undergo a joint sacrifice procedure at the time of the final follow-up. Choi et al. [20] retrospectively analyzed 31 patients with severe varus ankle osteoarthritis treated with SMOT at a mean age of 61.5 ± 7.3 years and concluded that SMOT was more effective in patients with varus ankle osteoarthritis, and that it had a significant effect on the improvement of patients' clinical symptoms despite the fact that the TT was not significantly corrected. It has also been studied in much younger patients. Kotlarsky et al. [23] performed supra-ankle osteotomy in seven patients with traumatic ankle varus deformity at a median age of 14 years (10–15 years), and the results demonstrated that SMOT can safely and effectively correct adolescent ankle varus deformity, and provided an anatomical correction of post-traumatic distal tibial varus deformity and restored ankle joint alignment. However, the number of patients included was limited, so more patients are needed to enrich the findings.

In this study, we confirmed that SMOT is equally good in correcting TAVD in adolescents, and we chose open medial wedge osteotomy over closed medial wedge osteotomy because it does not lead to shortening of the tibia in developing adolescents and the simple surgical approach makes it easier to accurately osteotomize the bone [23]. Our results showed that the mean preoperative TAS improved from 61.53 ± 3.74° in 32 patients to 88 ± 1.72° at 12 months postoperatively, the mean preoperative TT decreased from 2.25 ± 1.32° to 0.5 ± 0.57° at 12 months postoperatively, the mean preoperative TLS improved from 76.72 ± 0.21° to 79.34 ± 1.52° at 12 months postoperatively, all the radiographic outcomes were significantly improved, the mean preoperative AOFAS score improved from 65.5 ± 9.40 to 92.34 ± 4.00, the mean preoperative VAS score decreased from 2.44 ± 1.24 to 0.78 ± 0.75 at 12 months postoperatively, and the mean preoperative ROM of ankle improved from 50.16 ± 7.46 to 55.78 ± 4.77 at 12 months postoperatively, all of which were also significant improvements in clinical outcomes. Our findings were in good agreement with the results of SMOT treatment for adult patients with ankle arthritis in terms of radiographic outcomes and clinical results [24–26]; however, from the long-term follow-up results, the prognosis of adult patients with ankle arthritis had a strong correlation with the severity of preoperative ankle arthritis and their age [27, 28]; therefore, the authors concluded that SMOT is safer, more effective and has a better prognosis for adolescent patients.

There are some limitations to this study: Firstly, this was a retrospective study with a small number of patients included, which may have affected the accuracy of data collection and the accuracy of the results and did not provide the optimal angle of correction of the ankle for TAS, TT, and TLS. Secondly, some of the patients required additional fibular osteotomies to correct the deformity properly, and the impact of these procedures on clinical and radiographic outcomes is unknown. Third, without complete preoperative leg radiographs, possible deformities at the knee or hip level have remained undetected, and such deformities may influence decision making. In addition, the study lacked a control group. Finally, although the authors performed at least 1 year of clinical and imaging follow-up of the ankle, future studies should focus on collecting long-term outcomes and survival analyses to determine the effectiveness of SMOT.

## Conclusions

In conclusion, this study provides a preliminary discussion of the radiologic and clinical outcomes of SMOT for the treatment of TAVD in adolescents. Our study demonstrates that SMOT is effective in correcting TAVD and significantly improving ankle function in adolescents, and that it is an effective and successful method for restoring ankle joint congruence and normal hindfoot alignment.

## Data Availability

The datasets used and/or analyzed during the current study are available from the corresponding author on reasonable request.

## Abbreviations

SMOT: Supramalleolar osteotomy
TAVD: Traumatic ankle joint varus deformity
TAS: Tibial anterior surface angle
TT: Talar tilt angle
TLS: Tibial lateral surface angle
AOFAS: American Orthopedic Foot and Ankle Society
VAS: Visual Analogue Scale
ROM: Range of motion
